# Laccases with Variable Properties from Different Strains of *Steccherinum ochraceum*: Does Glycosylation Matter?

**DOI:** 10.3390/ijms20082008

**Published:** 2019-04-24

**Authors:** Olga A. Glazunova, Konstantin V. Moiseenko, Inna A. Kamenihina, Tatyana U. Isaykina, Alexander I. Yaropolov, Tatyana V. Fedorova

**Affiliations:** A.N. Bach Institute of Biochemistry, Research Center of Biotechnology of the Russian Academy of Sciences, Moscow 119071, Russia; mr.moiseenko@gmail.com (K.V.M.); innane@gmail.com (I.A.K.); superlilu277@gmail.com (T.U.I.); yaropolov@inbi.ras.ru (A.I.Y.)

**Keywords:** catalytic parameters, *Steccherinum ochraceum*, laccase, isoform, glycosylation

## Abstract

Laccases are blue multi-copper oxidases with an extensive number of actual and potential industrial applications. It is known that laccases from different fungal strains may vary in properties; however, the reason of this remains unclear. In the current study we have isolated and characterized seven laccases from different strains of *Steccherinum ochraceum* obtained from regions of central Russia. Although all seven laccases had the same primary sequences, there was a little variation in their molecular weights and thermostabilities. Moreover, statistically significant differences in laccases’ catalytic parameters of oxidation of phenolic substrates and ABTS were observed. After the deglycosylation of four selected laccases by Endo H and PNGase F, their affinities to pyrocatechol and ABTS became the same, suggesting a substantial role of *N*-linked glycosylation in moderation of enzymatic properties of laccases.

## 1. Introduction

Laccases (*p*-diphenol:dioxygen oxidoreductase, EC 1.10.3.2) are blue multi-copper oxidases that catalyze oxidation of a wide range of phenolic and non-phenolic aromatic compounds, coupled with a concomitant reduction of molecular oxygen to water [1,2]. Being environmentally friendly “green catalysts” with broad substrate specificity, laccases are proposed for an extensive number of actual and potential applications [3,4,5]. The applications of laccases include, but are not limited to: pulp and paper, pharmaceutical, food, cosmetic, and textile industries [6,7,8]; detoxification of environmental pollutants [9]; organic synthesis [10]; bioremediation [11]; bioconversion of agricultural and forestry residues [12]; enzymatic and immunochemical assays [13]; biosensor fabrication [14]; and nanobiotechnology [15].

For a long time, different basidiomycete fungi have been regarded as the main source of the biotechnologically relevant laccases [16,17]. Now it is well known that typical fungal genome can encode up to 17 laccase isozymes; post-translational modification of which (i.e., glycosylation) can produce even greater diversity of laccase isoforms [18,19]. Consequently, exploration of different laccase isoenzymes and isoforms from different sources and their physico- and bio-chemical characterization can help to establish a “natural library” of enzymes with distinct characteristics relevant to specific biotechnological applications.

In the last decade several reports demonstrated that different strains of the same species can produce laccases with different properties and complementary biochemical features [20,21]. However, all these reports did not directly address the cause(s) of observed variations. Up until recently, the main hindrance for such investigations was an absence of data about whole laccase multigene families for each investigated fungus. Without knowledge about amino acid sequences of all laccases encoded in the genome of a particular fungal species, it is very difficult to conclude whether laccases obtained from different strains are different isoenzymes or different isoforms of the same isoenzyme.

*Steccherinum* is a genus of basidiomycete fungi belonging to the *Steccherinaceae* family (order Polyporales). Some members of this family—*Antrodiella faginea*, *Junghuhnia nitida*, *Steccherinum murashkinskyi*, *Steccherinum ochraceum* and *Steccherinum bourdotii*–were previously reported as fungi with high laccase-producing capability [22,23,24]. Besides being very thermostable, laccases from these fungi have high catalytic efficiency towards different phenolic substrates (especially of syringyl-type) and dyes [22,25,26].

In this study seven novel laccases from seven strains of *Steccherinum ochraceum* were purified and biochemically characterized; the nucleotide sequences of genes encoding these laccases were determined; and the influence of glycosylation on their catalytic properties was assessed.

## 2. Results

### 2.1. Purification and Identification

In order to obtain sufficient for further comparative study amounts of laccases, seven strains of *S. ochraceum* were cultivated by a submerged method on a liquid glucose-peptone medium supplied with CuSO_4_ as an inducer. The cultural broth was collected at the 20–25th day of cultivation, when the laccase activity reached the maximum. As a result of the multi-step purification procedure, seven laccases—So3120, So3174, So2134, So3827, So3398, So3617 and So3622—from the corresponding strains of *S. ochraceum*—LE-BIN 3120, LE-BIN 3174, LE-BIN 2134, LE-BIN 3827, LE-BIN 3398, LE-BIN 3617 and LE-BIN 3622—were purified. The average yield was 20–30% and the purification factor was 40–60-fold. The specific activities of the purified isoenzymes were 150–210 U·mg^−1^.

The identification of the laccases was performed by MALDI-TOF/TOF mass-spectrometry. The *de novo* obtained amino acid sequences of peptides where compared with the amino acid sequences of the laccases encoded in the genome of *S. ochraceum* LE-BIN 3174 (version RWJN01000000). All peptide sequences showed the 100% identity to the laccase encoded by the gene model EIP91_000398; at the same time, identity to the other encoded in the genome laccases was less than 60%. Hence, all seven laccases obtained in this study are the products of the orthologous (to the EIP91_000398 gene of *S. ochraceum* LE-BIN 3174) laccase genes in the corresponding strains. Additionally, it should be noted that all these genes are orthologous to the *S. murashkinskyi laccase 2* gene, which encodes the only laccase from the fungi of the *Steccherinaceae* family that was simultaneously characterized at the levels of nucleotide and amino acid sequences (GenBank: JQ403278.1), protein 3D structure (PDB ID: 5E9N), and physicochemical and catalytic properties of the enzyme [23].

### 2.2. Comparison of Physicochemical and Catalytic Properties

**Figure 1:**
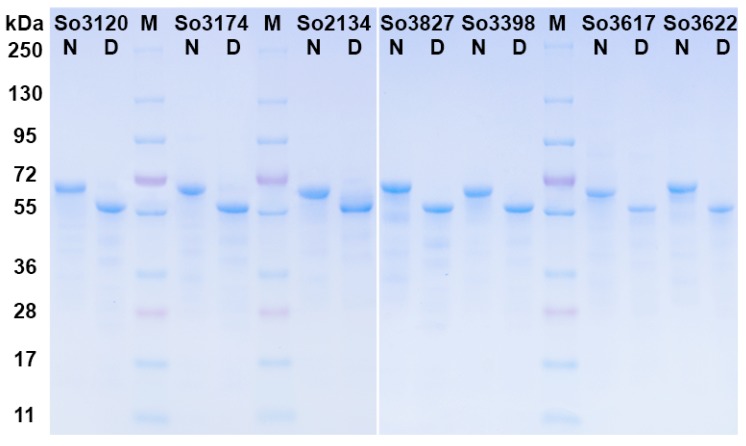
SDS-PAGE of *S. ochraceum* laccases. N: native laccase; D: deglycosylated laccase; M: molecular mass markers.

All purified laccases had a typical blue color, and their UV-Vis absorption spectra showed a pronounced band at 610 nm and a shoulder at 330 nm indicating the presence of Type 1 and Type 3 copper ions. Determined by SDS-PAGE molecular weights (MWs) of *S. ochraceum* laccases were slightly different: MWs of So3827 and So3622 were approximately 67 kDa; So3120 and So3174—66 kDa; and So2134, So3398, So3617—65 kDa (Figure 1). The p*I*s of obtained laccases determined by IEF-PAGE were approximately the same: 3.0 ± 0.1.

Regarding the half-time of inactivation (*τ*_1/2_) at 60 °С, the most stable laccases with *τ*_1/2_ around 900 min were So3120 and So3398; the less stable laccase with *τ*_1/2_ around 760 min was So3827; for the So3174, So2134, So3617 and So3622 *τ*_1/2_ were around 850, 880, 780 and 800 min, respectively. For all obtained laccases the melting temperature (*T*_max_) and enthalpy of denaturation (Δ*H*_cal_) determined by differential scanning calorimetry comprised 82.5–83.8 °С and 1265–1416 kJ/mol, respectively.

The kinetic parameters for oxidation of pyrocatechol, 2,6-DMP, guaiacol, syringaldazine and ABTS by all studied laccases are reported in Table 1. Generally, for all laccases *К*_м_ values decreased in the series guaiacol > pyrocatechol > ABTS > 2,6-DMP > syringaldazine, while *k*_cat_ values increased in the series 2,6-DMP < syringaldazine < guaiacol < ABTS < pyrocatechol. The detailed analysis of the obtained data revealed the presence of statistically significant variations for the *К*_м_ and *k*_cat_ values among different laccases tested with the same substrate. For the phenolic substrates the *К*_м_ values varied up to 80% and *k*_cat_—up to 60%, while for ABTS the *К*_м_ values varied up to 160% and *k*_cat_—up to 40%.

### 2.3. Identification of Gene Sequences

**Figure 2:**
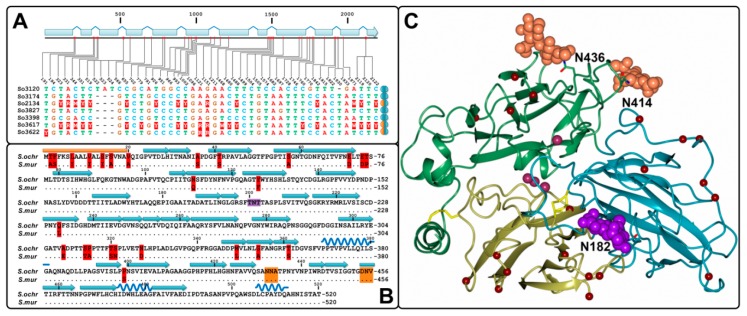
(**A**) Distribution of polymorphic sites within *S. ochraceum* laccase genes. Homozygotes and heterozygotes are marked by equally and differently colored semicircles, respectively. (**B**) Comparison of the amino acid sequences of *S. ochraceum* laccase(s) and *S. murashkinskyi* laccase 2. Signal peptide, beta strands and alpha helixes are depicted by gold rectangles, blue arrows and dark-blue springs, respectively. Amino acid substitutions are marked in red. Glycosylation sites Asn414 and Asn436 are marked in gold, and Asn182 in violet. (**C**) 3D structure model of *S. ochraceum* laccase(s). Cupredoxin-like domains are color-coded as follows: first domain—yellow, second dark—cyan and third—leaf green. Four copper ions are shown as purple spheres. Amino acid substitutions are depicted by red spheres. Carbohydrate moieties ((GlcNAc)_2_ retrieved from *S. murashkinskyi* laccase 2 structure) at the Asn414 and Asn436 sites are marked in gold, and at the Asn182 site—in violet.

The gene sequences that correspond to the studied laccase enzymes were PCR-amplified with the primers specific to the noncoding region of the gene model EIP91_000398 from the *S. ochraceum* LE-BIN 3174 genome. The nucleotide alignment of the obtained genes as well as the alignment of the encoded proteins demonstrated a remarkably high percentage of sequence identities. Although 46 single nucleotide polymorphisms (SNPs) were detected, almost 70% of these SNPs were located within introns (Figure 2A), and all SNPs in the exonic regions resulted in the synonymous mutations, which did not affect resulted amino acid sequence. The performed sequence analysis also demonstrated that 3 out of 7 investigated *S. ochraceum* strains were heterozygotes by the laccase gene under consideration. Hence, in our study we identified 10 laccase alleles in the population of *S. ochraceum*, all of which encoded 100% identical proteins.

The comparison of the obtained amino acid laccase sequence(s) from *S. ochraceum* strains with the sequence of laccase 2 from *S. murashkinskyi* showed the presence of 26 amino acid substitutions (Figure 2B). As it was shown by homology modelling using the *S. murashkinskyi* laccase 2 high resolution structure (PDB ID: 5E9N) as a template, all detected substitutions are located at the surface of the protein, mostly within the first cupredoxin domain (Figure 2C).

### 2.4. Assessment of Influence of Glycosylation

The deglycosylation of the obtained laccases with PNGase F and Endo H resulted in a decrease of their molecular weight by 8–10 kDa, leading to the same MW of 57 kDa for each (Figure 1). Consequently, the variation in MW of laccases could be attributed to the differences in their carbohydrate content. The circular dichroism (CD) spectra of the deglycosylated laccases almost completely matched those of the native ones.

For each laccase, the occupied glycosylation sites were determined by the analysis of the mass spectra of their native and deglycosylated forms (Table 2). In all seven native laccases two sites, Asn182 and Asn414, were glycosylated with carbohydrate moieties (Man)_X_(GlcNAc)_2_ where X varied from 5 to 8. The same carbohydrate moieties were detected at the Asn436 site in So3120, So3174, So3827, So3398 and So3622; however, no glycosylated peptides containing Asn436 were found in the mass spectra of So2134 and So3617. It can be assumed that this glycosylation site is absent in these two laccases, but it should be noted that corresponding peptides with unmodified Asn436 also were not found.

In the deglycosylated laccase samples no peptides with (Man)_X_(GlcNAc)_2_ moieties were found, but single *N*-acetylglucoseamine residues were still detected at the Asn182 and Asn414 sites. The exception was So3617 and So3622 for which no peptides containing Asn182 were detected. The masses corresponding to the peptides containing Asn436 modified with *N*-acetylglucoseamine were only detected in the mass spectra of deglycosylated So3827. The latter fact could be attributed to the overlap between a peak at *m*/*z* 1707 that corresponds to the Asn436 glycosylated with *N*-acetylglucoseamine and another stronger peak at *m*/*z* 1703.

**Figure 3:**
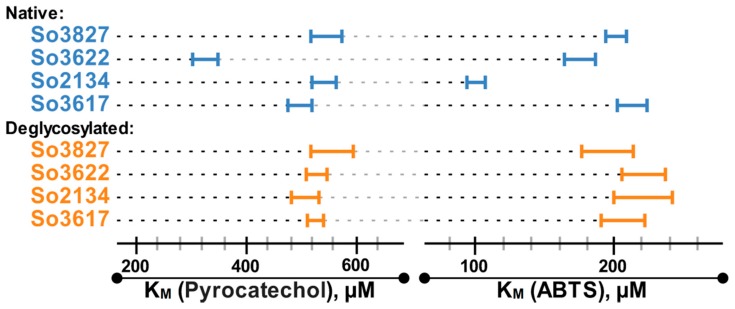
The comparison of the *K*_M_ values for pyrocatechol and ABTS oxidation by four selected laccases.

The assessment of the catalytic properties of 4 selected deglycosylated laccases, So2134, So3827, So3617 and So3622, using pyrocatechol and ABTS as substrates demonstrated a decrease in their catalytic activity (*V*_max_) after deglycosylation. Presumably, such decrease can be attributed to the partial inactivation of the enzyme, leading to the decrease in the active enzyme concentration. Importantly, after deglycosylation initial differences in the *K*_M_ values of the investigated laccases demolished (Figure 3); all deglycosylated laccases showed the same, within the margin of error, *K*_M_ values for both tested substrates (around 528 ± 25 for pyrocatechol and 208 ± 16 for ABTS). In comparison with the native samples the thermostability (*τ*_1/2_) of the deglycosylated laccases decreased 1.3 times for So3827 and 1.6–1.7 times for So2134, So3617 and So3622.

## 3. Discussion

In this study, we had purified and characterized seven laccase enzymes from seven different strains of *S. ochraceum* obtained from different regions of central Russia. As it was shown by mass spectrometry, all purified laccases are the products of orthologous genes (i.e., the same genes in different species). The detailed sequence analysis of these genes revealed that all point mutations in their coding regions were synonymous; hence, all purified laccases have 100% identical primary protein structures. Nevertheless, the obtained laccases showed differences in their physicochemical and catalytic properties. To account for this fact, the hypothesis about influence of glycosylation pattern on the enzymatic properties of purified laccases was advanced.

Although the differences of the molecular masses (on SDS-PAGE) of the purified laccases already suggested different degrees of their glycosylation, to fully corroborate our hypothesis three additional experiments were performed. Firstly, all laccases were deglycosylated; the identical MWs of deglycosylated laccases confirmed at the protein level that observed initial differences in the MWs of these laccases were attributed to the different degrees of their glycosylation and not to the other post translational modifications. Secondly, the fragmentation spectra of the native laccases were obtained and compared with those of the deglycosylated ones. As a result, the presence of three occupied glycosylation sites (Asn182, Asn414 and Asn436) was confirmed for five laccases, while for two other laccases only two sites (Asn182 and Asn414) were detected. Finally, the catalytic properties of the four selected deglycosylated laccases were measured using phenolic substrate pyrocatechol and nonphenolic ABTS. After deglycosylation *K*_M_ values of these laccases became practically the same (within the margin of error) for each tested substrate.

It is worth noting, that previously characterized laccase 2 from *S. murashkinskyi*, despite being very similar to the laccases obtained in this study, demonstrated significantly lower *K*_M_ value for guaiacol (1075 ± 50 µM) and slightly higher *K*_M_ value for ABTS (275 ± 27 µM) oxidations [23]. Considering that all 26 amino acid substitutions in laccase 2 from *S. murashkinskyi* are located far from the substrate binding pocket and unlikely affect catalytic properties of the enzyme (Figure 2C), described differences in the *K*_M_ values presumably can be attributed to the absence of Asn182 glycosylation site (which is present in all obtained *S. ochraceum* laccases) in its second cupredoxin domain (Figure 2B,C). Interestingly, the absence of Asn182 site in the *S. murashkinskyi* laccase 2 does not apparently influence its thermostability, which is similar (with *τ*_1/2_ at 60 °С equal to 890 min) to those of *S. ochraceum* laccases. However, the enthalpy of denaturation, which is associated with the proteins unfolding, was lower for *S. murashkinskyi* laccase 2 (893 kJ/mol), compared to *S. ochraceum* laccases (1265−1416 kJ/mol).

Hence, the performed experiments and indirect evidences from comparative analysis with *S. murashkinskyi* laccase 2 unambiguously demonstrate the importance of glycosylation pattern for moderation of enzymatic properties of laccases.

Currently, the most attention of researchers is focused on the nature of amino acid residues that surround the T1 copper binding sites of laccases and the organization of loops that form its substrate binding pocket [23,27,28,29]. In contrast, there are only two reports that directly address the role of glycosylation in fungal laccases. In [30] for *Pycnoporus sanguineus* laccase it was shown that deglycosylation resulted in increase of *K*_M_ values for phenolic substrates. In [31] for *Lentinus* sp. laccase it was shown that deglycosylated with Endo H laccase (with remained single *N*-acetylglucoseamine residues at each glycosylation site) exhibited the same *K*_M_ value for 2,6-DMP as native laccase, while the *K*_M_ value for ABTS decreases. However, both researches were conducted just for one laccase isoform from one fungal strain each.

In the present work we showed that the catalytic parameters of oxidation of 5 substrates could vary between the laccases produced by different fungal strains, and *N*-linked glycosylation could be the reason of these variations. Nowadays, modification of existing enzymes for their “fine tuning” is becoming a trend in the biotechnological industry. So, it is very important both from fundamental and practical points of view to further investigate the role of carbohydrate moieties attached to the laccase, since this can moderate enzyme functioning and should be taken into account during development of new and more effective biocatalysts.

## 4. Materials and Methods

The following fungal strains were obtained from the Collection of the Komarov Botanical Institute (LE-BIN), Russian Academy of Sciences (St. Petersburg): LE-BIN 3120, LE-BIN 3174, LE-BIN 2134, LE-BIN 3827, LE-BIN 3398, LE-BIN 3617 and LE-BIN 3622.

Cultivation was performed by submerged method using the glucose-peptone medium of the following composition (g·L^−1^): Glucose, 10.0; peptone, 3.0; KH_2_PO_4_, 0.6; K_2_HPO_4_, 0.4; ZnSO_4_ × 7H_2_O, 0.001; FeSO_4_ × 7H_2_O, 0.0005; MnSO_4_, 0.05; MgSO_4_ × 7H_2_O, 0.5; CaCl_2_, 0.5; supplied with 0.15 g·L^−1^ CuSO_4_ as described in [32].

Laccase purification and characterization (including determination of molecular weight; p*I*; thermostability, *τ*_1/2_; melting point, *T*_max_; and enthalpy of denaturation, Δ*H*_cal_) procedures were identical to those described in [22]. Laccase activity was measured spectrophotometrically with pyrocatechol as a substrate (ε_410_ = 740 cm^−1^·M^−1^). One unit of activity was defined as μM of product formed per min by 1 µg·L^−1^ of enzyme. CD-spectra were recorded with Сhirascan spectrometer (Applied Photophysics, Leatherhead, UK) in 20 mM potassium-phosphate buffer at 20 °C.

Kinetic constants were determined spectrophotometrically using a Lambda 35 spectrophotometer (PerkinElmer, Waltham, Massachusetts, USA) at 25 °C in 0.1 M McIlvaine buffer (pH 4.5) as described in [23].

The laccases were deglycosylated by the overnight treatment with PNGase F and Endo H (Sigma, Stenheim, Germany) in 0.05 M potassium-phosphate buffer (pH 6.5) at 37 °C according to manufacturer’s instructions. Deglycosylation was confirmed by SDS-PAGE.

For the mass spectrometry analysis the laccase bands obtained by SDS-PAGE were cutted, the gel samples were digested with trypsin (Promega, Madison, WI, USA), and the resulting peptides were spotted on a MALDI target plate. The mass spectra were obtained using Bruker Ultraflex II MALDI TOF/TOF mass spectrometer (Bruker Daltonics, Bremen, Germany). The fragmentation spectra were obtained using the tandem mode of the device, and the accuracy on measurement of fragmented ions was at least 2 Da. The mass spectra data were processed using the FlexAnalysis 3.3 program (Bruker Daltonics, Germany). The search for proteins corresponding to MALDI-TOF/TOF MS data was carried out with Mascot Peptide Mass Fingerprint in the local database containing protein sequences from the genome of *S. ochraceum* LE-BIN 3174 (version RWJN01000000). The search with combined data of the peptide masses and peptide fragmentation was performed by Biotools 3.2 (Bruker Daltonics, Germany).

Identification of the laccase gene sequences was performed using PCR primers: FP (5′-ATCAGCTTCACATCTAGGCA-3′) and RP (5′-CTTTACGTTCAAGTAGCCG-3′). The PCR amplification was performed with the Encyclo PCR kit (Evrogen, Moscow, Russia) under the following conditions: 1 cycle of 2 min at 95 °С; 28 cycles of (20 s at 95 °С, 30 s at 56 °С, and 2 min at 72 °С); 1 cycle of 3 min at 72 °С. Obtained PCR products of ~2200 bp were purified from the 1.4% agarose gel using the QIAquick Gel Extraction Kit (Qiagen, Hilden, Germany) and sequenced by the standard Sanger sequencing method.

Homology modeling of *S. ochraceum* laccase was performed using SWISS-MODEL protein structure homology-modeling server (http://swissmodel.expasy.org/) [33]. The crystal structure of *S. murashkinskyi* laccase (PDB ID: 5E9N, 95% of identity with *S. ochraceum* laccase) was used as a template.

Obtained *К*_м_ and *k*_cat_ values were statistically analyzed with ANOVA, followed by the Tukey’s HSD post hoc tests (*p* < 0.05). Whenever appropriate, data are represented by the mean ± standard deviation (SD).

## Figures and Tables

**Table 1:** The kinetic parameters for oxidation of phenolic substrates and ABTS by laccases.

Laccase	Kinetic Parameters	ABTS	Pyrocatechol	Guaiacol	2,6-DMP	Syringaldazine
So3120	*K*_M_, µM	175 ± 23	497 ± 31	2665 ± 216	17 ± 3	5.4 ± 0.8
	*k*_cat_, s^−1^	325 ± 28	406 ± 24	165 ± 20	110 ± 14	105 ± 12
	*k*_cat_/*K*_M_, s^−1^·mM^−1^	1857 ± 290	817 ± 70	62 ± 9	6471 ± 1273	19,444 ± 3617
So3174	*K*_M_, µM	81 ± 9	576 ± 86	2230 ± 205	20 ± 2	3.8 ± 0.6
	*k*_cat_, s^−1^	240 ± 21	373 ± 27	152 ± 13	101 ± 9	104 ± 11
	*k*_cat_/*K*_M_, s^−1^·mM^−1^	2963 ± 416	648 ± 108	68 ± 9	5050 ± 755	27,368 ± 5091
So2134	*K*_M_, µM	100 ± 6	540 ± 22	2060 ± 168	21 ± 2	3.7 ± 0.5
	*k*_cat_, s^−1^	254 ± 23	400 ± 35	221 ± 20	166 ± 14	91 ± 7
	*k*_cat_/*K*_M_, s^−1^·mM^−1^	2540 ± 276	741 ± 72	107 ± 13	7905 ± 1034	24,595 ± 3929
So3827	*K*_M_, µM	200 ± 7	545 ± 26	3565 ± 363	24 ± 3	6.7 ± 0.9
	*k*_cat_, s^−1^	276 ± 31	394 ± 37	237 ± 22	165 ± 15	113 ± 12
	*k*_cat_/*K*_M_, s^−1^·mM^−1^	1380 ± 162	723 ± 76	66 ± 9	6875 ± 981	16,866 ± 2872
So3398	*K*_M_, µM	209 ± 27	495 ± 41	2426 ± 225	17 ± 3	5.6 ± 0.8
	*k*_cat_, s^−1^	281 ± 26	450 ± 40	262 ± 24	152 ± 12	119 ± 11
	*k*_cat_/*K*_M_, s^−1^·mM^−1^	1344 ± 214	909 ± 110	108 ± 14	8941 ± 1516	21,250 ± 3565
So3617	*K*_M_, µM	212 ± 10	495 ± 21	2383 ± 257	22 ± 2	5.2 ± 0.6
	*k*_cat_, s^−1^	228 ± 21	340 ± 31	197 ± 20	134 ± 11	89 ± 10
	*k*_cat_/*K*_M_, s^−1^·mM^−1^	1075 ± 111	687 ± 69	83 ± 12	6091 ± 836	17,115 ± 2691
So3622	*K*_M_, µM	174 ± 11	323 ± 25	2717 ± 272	23 ± 3	5.5 ± 0.7
	*k*_cat_, s^−1^	252 ± 20	365 ± 34	218 ± 17	140 ± 18	83 ± 7
	*k*_cat_/*K*_M_, s^−1^·mM^−1^	1448 ± 147	1130 ± 137	80 ± 10	6087 ± 1163	15,091 ± 2213

**Table 2:** *N*-glycosylation sites and glycan structures found in the native and deglycosylated *S. ochraceum* laccases using MALDI-TOF/TOF mass spectrometry data.

Laccase	*N*-Glycosylation Sites		
	Asn182	Asn414	Asn436
So3120 N *	(Man)_5–8_(GlcNAc)_2_	(Man)_6–8_(GlcNAc)_2_	(Man)_6–8_(GlcNAc)_2_
So3120 D *	GlcNAc	GlcNAc	n.d. **
So3174 N	(Man)_5–7_(GlcNAc)_2_	(Man)_6–8_(GlcNAc)_2_	(Man)_5–8_(GlcNAc)_2_
So3174 D	GlcNAc	GlcNAc	n.d.
So2134 N	(Man)_5–7_(GlcNAc)_2_	(Man)_5–8_(GlcNAc)_2_	n.d.
So2134 D	GlcNAc	GlcNAc	n.d.
So3827 N	(Man)_5–7_(GlcNAc)_2_	(Man)_6–8_(GlcNAc)_2_	(Man)_7–8_(GlcNAc)_2_
So3827 D	GlcNAc	GlcNAc	GlcNAc
So3398 N	(Man)_5–7_(GlcNAc)_2_	(Man)_6–8_(GlcNAc)_2_	(Man)_5–7_(GlcNAc)_2_
So3398 D	GlcNAc	GlcNAc	n.d.
So3617 N	(Man)_5–7_(GlcNAc)_2_	(Man)_6–8_(GlcNAc)_2_	n.d.
So3617 D	n.d.	GlcNAc	n.d.
So3622 N	GlcNAc, (Man)_5–7_(GlcNAc)_2_	(Man)_6–8_(GlcNAc)_2_	(Man)_7–8_(GlcNAc)_2_
So3622 D	n.d.	GlcNAc	n.d.

* N: native laccase; D: deglycosylated laccase; n.d. **: peptides were not detected.

## Abbreviations

2,6-DMP	2,6-dimetoxyphenol
ABTS	2,2′-azino-bis(3-ethylbenzothiazoline-6-sulphonic acid)
GlcNAc	*N*-acetylglucoseamine
Man	Mannose
PDB	Protein Data Bank
SNP	Single Nucleotide Polymorphism
